# Scientific validation of medicinal plants used by Yakkha community of Chanuwa VDC, Dhankuta, Nepal

**DOI:** 10.1186/s40064-016-1821-5

**Published:** 2016-02-24

**Authors:** Bimala Subba, Chitranjan Srivastav, Ram Chandra Kandel

**Affiliations:** Central Department of Chemistry, Tribhuvan University, Kirtipur, Kathmandu, Nepal

**Keywords:** *Yakkha*, Ethnic group, Antibacterial, Medicinal plants, Antioxidant

## Abstract

Ethnobotanical knowledge is important among tribal people, but much of the information is empirical due to the lack of scientific validation. The purpose of this study was to document the medicinal plants used by an ethnic group (*Yakkha*) at Chanuwa VDC of Dhankuta district in Nepal and to validate scientifically in the use of plants based on results of phytochemical, antimicrobial and antioxidant property analyses and available literature reports. Data were collected through interviews of the *Yakkha* people with the help of a semi-structured questionnaire and the guided field walk method. A total of 30 different medicinal plants were recorded along with their vernacular names (for few plants) used by the *Yakkha* community’s people. Literature review reveals that most of the plant species described herein have also been used in other countries, too. Among 30 plants selected for this study methanol extract of five ethno-medicinal plants viz., *Dendrocnide sinuata*, *Solanum anguivi*, *Pogostemon cablin*, *Boehmeria platyphylla* and *Clerodendrum trichotomum* and ethanol extract of *C. trichotomum* were subjected for antibacterial and antioxidant properties. The antimicrobial activities were measured using the paper disc diffusion method. The antioxidant properties of plants were measured by DPPH and FRAP reduction assay. Among all extracts, ethanol extract of *C. trichotomum* and methanol extract of *B. platyphylla* displayed the highest antibacterial and antioxidant activities, respectively.

## Background

Natural resources have been a huge and diversified chemical bank. Of large number of modern drugs isolated from natural sources, identification of significant number of plant-based drugs has been guided by their traditional use as medicine (Newman and Cragg 2007; Balunas and Kinghorn 2005; Zhang 2002). In recent years much attention has been paid to plants as a source of therapeutic agents due to their medicinal value (e.g. antimicrobial and antioxidants properties), the reasonable cost, easy availability, and relatively lower incident of other adverse effects compared to synthetic pharmaceuticals. In other words, this is due to increased awareness of the limited ability of synthetic pharmaceutical products to control major diseases and the need to discover new molecular structures as lead compounds from the plant kingdom (N’guessan et al. 2007; Thomas 1997).

In this scenario, Nepal is a storehouse of natural medicinal plants. It is a multiethnic nation. Ethnomedicine is the study of medicinal plants used by ethnic groups or indigenous societies for the treatment and cure of various ailments. The ethnic groups are rich in indigenous knowledge and it is deeply rooted in their tradition and cultures. Ethno botanical knowledge on various plants acquired by their self-experience is rapidly eroding due to the lack of communication between the scientific community and indigenous communities (Bhattarai et al. 2006, 2009). A particular ethnic group may be knowledgeable on one or a few such medicinal properties of a plant but not about all the plants. As a result, there is a curcial need to document such ethnomedicinal or traditional medicinal practices, before they get lost either due to ethnic extinctions or extinctions of an ethnomedicinally used plant species. The phytochemical research based on ethnopharmacological information is considered as an effective approach in the discovery of new agents from higher plants (Chen et al. 2008; Duraipiyan et al. 2006). Ethnomedicinal documentation combined with screening of their antibacterial and antioxidant properties is one convincing way to discover newer drugs, especially in this age of emergence of new diseases (like Ebola) or multi drug resistant pathogens or disease related to oxidative stress such as cancer and Alzheimer. The effectiveness of phytochemicals in the treatment of various diseases may lie in their antioxidant effects because oxidative stress is associated with pathogenic mechanisms of many diseases including atherosclerosis, neurodegenerative diseases, cancer, diabetes and inflammatory diseases, as well as aging processes. Antioxidants can delay, inhibit or prevent the oxidation of oxidizable materials by scavenging free radicals and diminishing oxidative damage (Duracková 2010).

While some ethnopharmacological studies have been conducted in Nepal, many parts of the country remain unexplored (Bhattarai et al. 2006; Rajbhandari 2001). Few studies have attempted to evaluate the bio-efficacy of traditionally used medicinal plants (Shrestha and Dhillion 2003; Kunwar et al. 2009). In this context, this study documents medicinal plants used by one ethnic group (*Yakkha*) residents in Chanuwa VDC, Dhankuta district, Nepal and screening the five plants i.e. *Dendrocnide sinuate* (Bhum) Chew, *Solanum anguivi* Lam., *Pogostemon cablin* (Blanco) Benth, *Boehmeria platyphylla* Buch.-Ham.ex D.Don and *Clerodendrum trichotomum* Thunb for phytochemical, antimicrobial and antioxidant properties.

### Study area

**Fig. 1:**
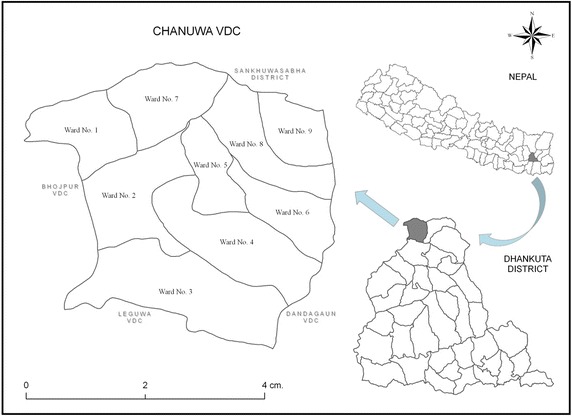
Location of the study area is Dhankuta district of East Nepal

Nepal is a multiethnic and multilingual nation with more than 60 different ethnic groups speaking about 75 languages in Nepal (Shrestha 1998). Dhankuta district lies between 26°59′ north latitude and 87°20′ east longitude of Nepal. Its total area is about 891 km^2^ and the average temperature ranges from 4 to 31 °C. The altitude ranges from 300 to 2500 m from the sea level. The major ethnic people of this district are Tamang, Sherpa, Chhetri, Brahimin, Newar, Magar, Pariyar, Biswakarma, Yakkha, Rai and Limbu. These ethnic people have a common Nepali dialect, but some ethnic people such as Tamang, Newar, Magar, Limbu, Rai and Yakkha have their own dialects. Present ethnomedicinal study is carried out on one least populated ethnic group (*Yakkha*) in Chanuwa VDC of Dhankuta district (Fig. 1).

## Results and discussion

### Plant species used in traditional medicine

**Fig. 2:**
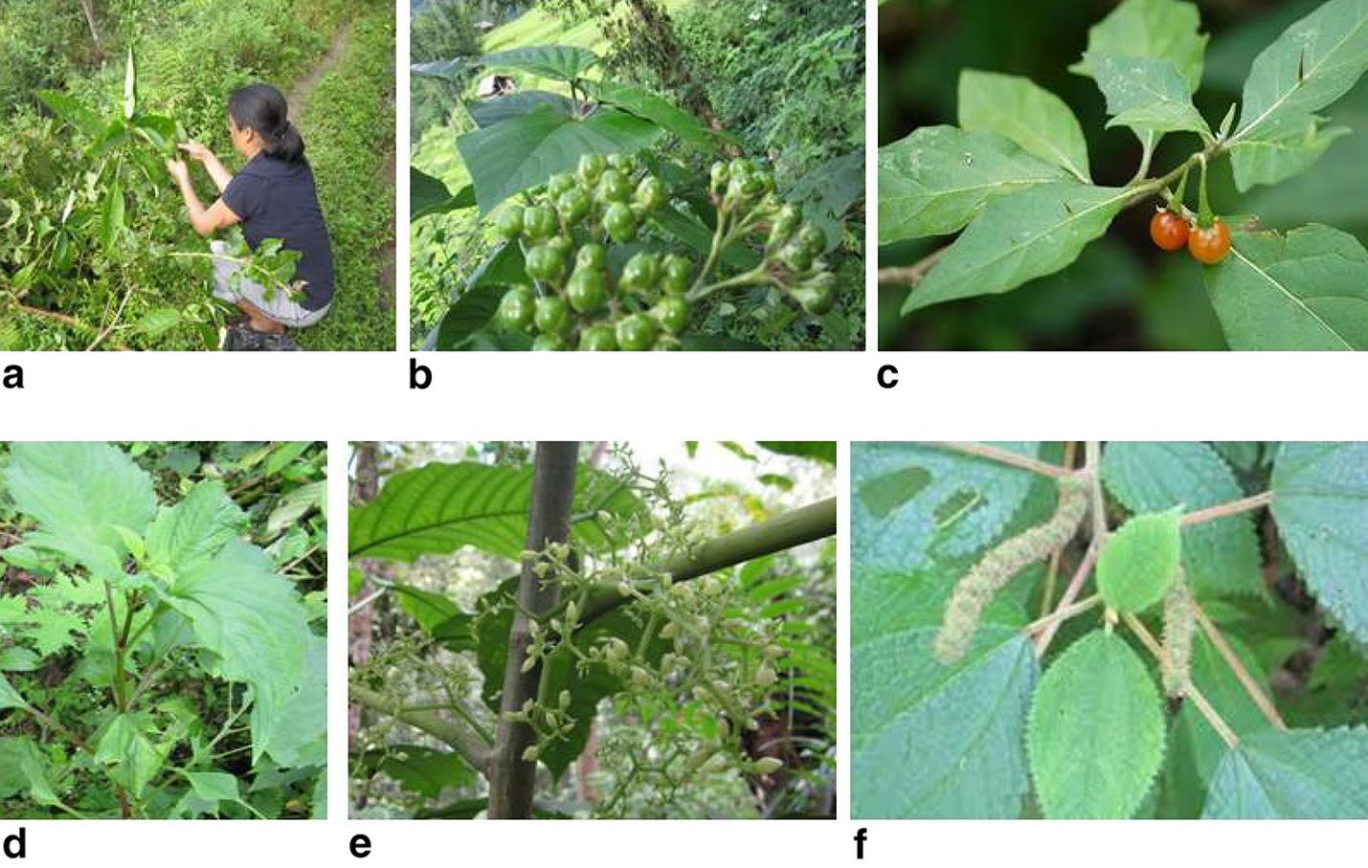
Collected plants for scientific evaluation. **a** Picture of collecting of plants, **b** *Clerodendrum trichotomum* (*Lopche*), **c** *Solanum anguivi* (*Khikwaringba*), **d** *Pogostemon cablin*, **e** *Dendrocnide sinuata* (Moringe), **f** *Boehmeria platyphylla*

The present finding reveals that the *Yakkha* people of the study area have good knowledge on the use of different medicinal plant species for their own local health care system. It is found that they make the uses of some 30 species of medicinal plants belonging to 25 families with curing different ailments/diseases by using their own indigenous knowledge system. The plants that were available at the season of our field visit were randomly selected for scientific evaluation (Fig. 2). The scientific evaluation of remaining plants reported here is still going on as a part of our continued research.

Plant species are arranged in alphabetical order, mentioning the botanical names. Family names are kept in parenthesis, vernacular names are kept along with a brief description of preparation and use. The vernacular names are abbreviated as Nepali (N) and *Yakkha* (Y).

*Acorus calamus* L. (Acoraceae); ‘Bojho’ (N). Juice of rhizome is used to treat certain skin scabies. Rhizome is chewed in case of appetizer and sore throat, too.

*Ageratina adenophora* (Spreng.) R.M. King & H. Rob. (Asteraceae); ‘Banmaara’ (N), The leaf paste is used to stop bleeding from the fresh cuts and wounds. Juice of root is useful for fever treatment. A paste of the leaf is applied to cure boils and it is also used to treat eyes insomnia.

*Alternanthera sessilis* (L.) R.Br. (Amaranthaceae); ‘Bhiringe jhar’ (N). Root juice is used to cure bloody dysentery.

*Artemisia vulgaris* L. (Asteraceae); ‘Tite pati’ (N); ‘Pati’ (Y). Its leaf paste is used to cure skin scabies and to stop the nose bleeding.

*Aglaomorpha coronans* Wall. ex Mett. (Polypodiaceae); ‘Kamaru’ (Y). Its root’s juice is used to cure diarrhea and constipation.

*Bauhinia variegate* L. (Fabaceae); ‘Koiralo’ (N). A decoction of flower is used to cure dysentery and mouth sores.

*Boehmeria platyphylla* Buch.-Ham.ex D.Don (Urticaceae); ‘Kamle’ (N). Root juice paste is used quickly to stop bleeding in minor cut and wounds. A decoction of the plant is given to livestock for diarrhea and dysentery. A paste of the root is used to treat cattle wounds and cuts.

*Cissus repens* Thwaites. (Vitaceae); ‘Jogilahara’ (N). Plant is used in boils and abscesses or maturation. It is applied in sloughing and foetid ulcers.

*Clerodendrum trichotomum* Thunb. (Lamiaceae); ‘Lapche’ (Y). Young leaves are mixed up with rice and cooked as vegetables soup which is commonly taken as blood pressure lowering food item by the *Yakkha* community. Shoots are also preserved in the form of sundry powder. Powdered shoots are taken orally on daily basis to manage high blood pressure.

*Dendrocnide sinuate* (Bhum) Chew. (Urticaceae); ‘Moringe’ (Y). A decoction of root is used to cure jaundice.

*Drymaria cordata* (L.) Willd. ex Schult. (Caryophyllaceae); ‘Abijaalo’ (N); ‘Wakerkma’ (Y). This plant is used to avoid coldness.

*Lyonia ovalifolia* (Wall.) Drude. (Ericaceae); ‘Angeri’ (N). Crushed leaves are used in Scabies. Young leaves are poisonous to cattle.

*Equisetumramosissimum* Desf. (Equisetaceae); ‘Chasme jhar’ (N). Whole plant is used as cooling medicine and it is given in gonorrhea. Its root is used in diarrhea and dysentery for babies and making amulets.

*Hedyotis scandens* Roxb. (Rubiaceae); ‘Dudhelahara’ (N). Plant is used in eye diseases and in troubles following child birth. Root is used for sprain. Root paste is used in indigestion and plant paste is used for treatment of peptic ulcer.

*Hibiscus lampas* Cav. (Malvaceae); ‘Bankapas’ (N). Root and fruits are used in gonorrhea and syphilis. Decoction of roots is taken in case of jaundice treatment. Root paste is applied on boils.

*Jatropha* sect.*Curcas* (Adans.) Griseb. (Euphorbiaceae); ‘Kadam or Sajiban’ (N). Latex paste is applied on wounds 2–3 times a day until recovery. Plant juice is a well known purgative and used in whitlow, convulsion, syphilis, neuralgia, dropsy, anasarca and pneumonia. Leaves are galactogogue, rubefacient, insecticidal and they are used in tumors and scabies.

*Lygodium japonicum* (Thunb.) Sw. (Lygodiaceae); ‘Sakhewa hiruwa’ (Y). Crushed leaves are used in fresh cuts and wounds for the fast recovery.

*Mentha* subsect. *Spicata* L. (Lamiaceae); ‘Pudina’ (N). Leaf juice is given to treat vomiting and gastric disorder. Leaves are also chewed for appetizer and boils on the tongue. Leaves are used to prepare pickle.

*Lindera neesiana* (Wall.ex Nees) Kurz. (Lauraceae); ‘Siltimur’ (N). This plant is used to treat fever, stomach disorder and for the treatment of animals.

*Morus alba* L. (Moraceae); ‘Kambu’ (N); ‘Magmeru’ (N). Bark of the plant is used as astringent, carminative and antiseptic. Decoction of bark is useful in cure of asthma, lungs infection, chronic bronchitis, diarrhea and dysentery.

*Ocimum sanctum* L. (Lamiaceae); ‘Tulsi’ (N). Leaves are remedy for the treatment of headaches caused due to sinusitis, allergies, cold or even migraines and tonsils.

*Osyris wightiana* Wall. ex Wight. (Santalaceae); ‘Nundhiki’ (N). Infusion bark of this plant is given to women after delivery to stop bleeding. Infusion of leaves is emetic. Leaves are used as tea.

**Fig. 3:**
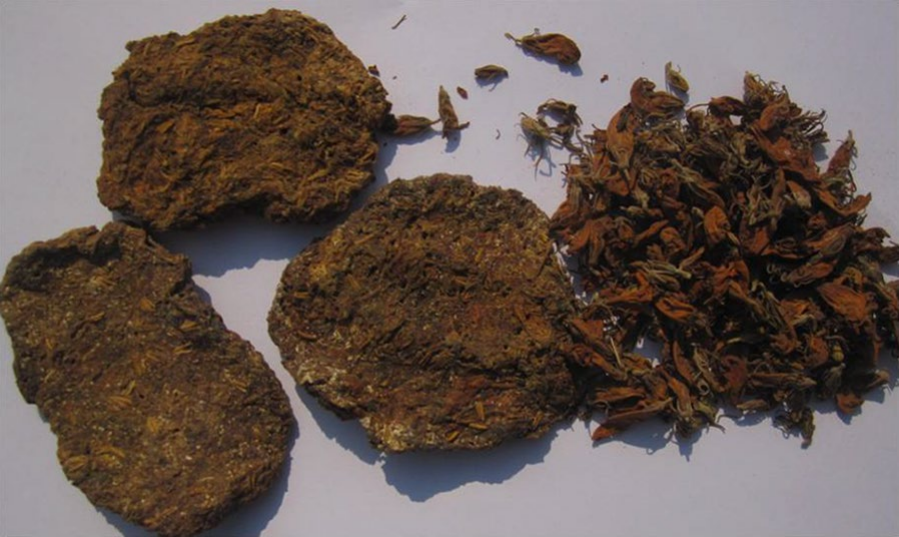
Cake of flower and dry flower of *P. thyrsiflormis*

*Phlogacanthus thyrsiflormis* Mabb. (Acanthaceae); ‘Okmilang’ (Y). The flower with young inflorescence is mixed up with rice (Bhat) and fried with vegetable in oil to prepare a special item called ‘teete’ which is commonly taken as favourite food item by the *Yakkha* community. Young shoots and flowers are also crushed and used to prepare sundry cakes (Fig. 3). Leaf juice is used in high blood pressure, cough, asthma and rheumatism. The flowers are sold in the markets which cost 500–600 rupees per kg.

*Psidium guajava* L. (Myrtaceae); ‘Amba’ (N); ‘Belti’ (Y). Leaf buds are chewed to treat fever and headaches. Bark juice is given in loss of appetite and dysentery. Leaves juice is taken to treat bowels, wounds and ulcers. A decoction of the leaves is applied in cholera, diarrhea and indigestion. A paste of leaf is used for treatment of rheumatism, cuts and wounds.

*Pogostemon cablin* (Blanco) Benth. (Lamiaceae); ‘Setojhar’ (N). Roots are remedy for haemorrhage and antidote. Fresh leaves are styptic, bruised and applied as cataplasm to clean wound and promote healthy granulation.

*Schima wallichii* Choisy. (Theaceae); ‘Chilaunee’ (N); ‘Yangsingbu’ (Y). The paste of the fruit is used against poisonous spiders bite.

*Solanum anguivi* Lam. (Solanaceae); ‘Sanobihi’ (N); ‘Khikwaringba’ (Y). Fruits are cooked as vegetable, pickled in different ways and eat against diabetic and high blood pressure. Crushed fruits are applied on head to get relief from headaches.

*Tectaria coadunata* (J. Sm.) C. Chr. (Tectariaceae); ‘Kalo niuro’ (N). The rhizome of the plant is used against stomach pain, gastrointestinal disorders, eradication of worms in children and jaundice.

*Terminalia chebula* Retz. (Combretaceae); ‘Harro’ (N); ‘Tungwa’ (Y). It is used in treatment of cough and sore throat.

*Urtica dioica* L. (Urticaceae); ‘Sisnu’ (N); ‘Chhiwa’ (Y). Young shoots are cooked as vegetable and eat as curry by poor people but it is good for curing blood pressure of all the people. The paste of root is used in dog bites, too.

### Phytochemical screening

The phytochemical screenings of methanol extracts of selected plants were conducted on the basis of procedure given by Prof. I. Culie. The results obtained are tabulated below (Table 1). The qualitative phytochemical screenings of the crude methanolic extract were conducted. The results indicated the presence of reducing compounds and flavonoids in all the extracts. The glycosides were absent in *Dendrocnide sinuata* while the polyphenols were absent in *D. sinuata* and *S. anguivi*. The phytochemical screening of the plant studied in the present study has not been reported previously. The result is well supported as several flavonoids, diterpenes, sterols and phenylpropanoid glycosides have been isolated from *C. trichotomum* (Kim et al. 2001; Kato et al. 1971; Okigawa et al. 1970; Kawano et al. 1967), and methanol extract of *S. anguivi* has been reported to contain phenolic acids such a gallic acid, chlorogenic acid, caffeic acid and the flavonoid contents such as rutin by HPLC fingerprinting (Elekofehinti et al. 2013).

**Table 1:** Phytochemical constituents of analyses plants

Plants’ name	Reducing compounds	Glycosides	Polyphenols	Quinones	Alkaloids	Flavonoid	Saponin
*D. sinuate*	+ +	− −	− −	− −	+ +	+ +	− −
*S. anguivi*	+ +	+ +	− −	− −	− −	+ +	− −
*P. cablin*	+ +	+ +	+ +	− −	+ +	+ +	− −
*B. platyphylla*	+ +	+ +	+ +	+ +	− −	+ +	− −
*C. trichotomum*	+ +	+ +	+ +	+ +	− −	+ +	− −

− = Absent, ++ = present

Various studies have revealed flavonoids to inhibit or kill many bacterial strains. Similarly, phenolic compounds have been reported to possess biological properties such as anti-aging, anticancer, anti-inflammatory, etc. Many studies have described the antioxidant properties of phenolic compounds as well. While glycosylated products are known to possess anti-bacterial, anti-tumor and anti-oxidative activities, saponins are known as good things to enhance antibody production.

The present study also indicated the presence of various phytochemicals in different extracts. Most of the extracts have reflected the presence of polyphenols, glycosides, alkaloids and flavonoids. Thus, these phytochemicals can be attributed to the potential antibacterial and antioxidant properties in the tested samples.

### Antibacterial activity

In this bioassay the activity of the crude extracts against different micro-organisms was studied. The results are tabulated below (Table 2).

**Table 2:** Antibacterial activities of methanol extract of the test plants

Plants	Name of microorganism	Mean zone of inhibition (mm)^a^	
		50 (μg/disc)	100 (μg/disc)
*D. sinuate*	*E. coli*	6 ± 0.25	6 ± 015
	*S. aureus*	7 ± 0.35	7 ± 0.2
	*B. subtilis*	8 ± 0.2	9 ± 0.25
	*S. typhi*	6 ± 0.30	7 ± 0.2
*S. anguivi*	*E. coli*	na	na
	*S. aureus*	na	na
	*B. subtilis*	8 ± 0.2	9 ± 0.2
	*S. typhi*	6 ± 0.1	7 ± 0.2
*P. cablin*	*E. coli*	7 ± 0.20	8 ± 0.25
	*S. aureus*	na	na
	*B. subtilis*	na	na
	*S. typhi*	8 ± 0.35	9 ± 0.25
*B. platyphylla*	*E. coli*	na	na
	*S. aureus*	7 ± 0.1	8 ± 0.25
	*B. subtilis*	na	na
	*S. typhi*	6 ± 0.2	7 ± 0.2
*C. trichotomum*	*E. coli*	na	na
	*S. aureus*	6 ± 0.26	7 ± 0.81
	*B. subtilis*	na	na
	*S. typhi*	na	na

*na* not active

^a^Mean zone inhibition of three replicates

The leaf extract of *P. cablin* showed a moderate activity against *E. coli* and *S. typhi*, but no activity has been noted against *B. subtilis* and *S. aureus*. Its strong inhibitory effect in vitro against *S. aureus*, *P. vulgaris*, *K. pneumonia* and *E. coli* from ethanolic extract has been reported (Subba and Basnet 2014). *D. sinuata* showed a moderate activity against all the tests of micro-organisms. *S. anguivi* and *B. platyphylla* showed moderate activity against the *S. aureus* and *S. typhi*, but no activity against *E. coli* and *B. subtilis*. The antibacterial activity of *D. sinuata*, *S. anguivi* and *B. platyphylla* has not been reported previously. In overall, the result obtained here revealed the extracts being active against both gram-positive bacteria and gram-negative bacteria. In the present study methanolic extract of *C. trichotomum* showed moderate activity against *S. aureus*, but no activity against all other test organisms has come. It has been reported that 22-dehydroclerosterol and β-amyrin isolated from the methylene chloride (MC) fraction of *C. trichotomum* revealed moderate antibacterial effects against *E. coli*, *S. aureus*, and *H. pylori* (Choi et al. 2012). Successive isolation of phytochemicals from plant material is largely dependent on the type of solvent used in extraction procedures. Different extracts are used to test the antimicrobial properties of medicinal plants. The traditional healers use primarily water as solvents. But a number of researchers found that plant extracts prepared with methanol and ethanol as solvent provided more consistent antimicrobial activity (Parekh and Chanda 2007). Alcohol extracts provide a more complete extraction including less polar compound, and many of these extracts have been found to possess antimicrobial properties. The ethanol solvent is known for its ability to extract more antimicrobials and antioxidants from plants including tannins, polyphenols, terpenoids, saponins, xanthoxylines, totarol, quassinoids, lactones, flavones and phenones; while the water extracts could contain only anthocyanins, starches, tannins, saponins, polypeptides and lectins (Cowan 1999). Hence, in the present study ethanolic extract of *C. trichotomum* was also subjected for antibacterial test against *S. aureus*, *E. coli*, *Proteus vulgaris* and *Klebsiella pneumonia*. The results are tabulated below (Table 3).

**Table 3:** Antibacterial activities of ethanol extract of *C. trichotomum*

Name of microorganism	Mean zone of inhibition (mm)^a^	
	50 (μg/disc)	100 (μg/disc)
*E. coli*	16 ± 0.5	16 ± 0.5
*S. aureus*	16 ± 0.26	16 ± 0.2
*P. vulgaris*	20 ± 0.15	20 ± 0.21
*K. pneumonia*	15 ± 0.16	20 ± 0.46

^a^Values are mean zone inhibition ± SD three replicates

Medicinal plants generally contain several compounds which may serve as an alternative, effective, cheap, and safe treatment for common bacterial infections. In the present study, ethanolic extract of *C. trichotomum* have shown the broad spectrum activity. It inhibited the growth of both gram positive and gram negative bacteria. Ethanol extracts of *C. trichotomum* exhibited a higher degree of antimicrobial activity as compared with methanol extracts fractions. This finding is correlated with the medicinal preparations that use rum and liquor to extract the active plant components. Hence, ethanol in general represents a better choice for the extraction of antibacterial agent from *C. trichotomum* and *P. cablin*.

The literature review revealed that the antimicrobial activity of higher plants depends upon the presence of secondary metabolites such as alkaloids, tannins, flavonoids and other phytochemicals. In many cases phytochemicals can be more effective than chemically synthesized pure compounds as a result of synergistic action of complex mixture of compounds. The constituents may interact with multiple molecular targets at a time and thus makes it more difficult for target microorganisms to develop resistance. Thus, in accordance to the literature, the present study also revealed various phytochemicals in different extracts and some of the test extracts in the current work have exhibited significant antibacterial activity. Hence, there are chances that some of test extracts in present study may find their use as antibacterial agents in the days to come. Few of the species in present study have showed promising antibacterial property, which demonstrates their implication in traditional remedies and inaccessible residents.

### Antioxidant activity

#### DPPH assay

The antioxidant properties of the plant extracts and standard ascorbic acid were assessed by their DPPH radical scavenging activity. The antioxidant activity of crude methanolic extract of only *P. cablin*, *B. platyphylla*, *C. trichotomum* and ethanol extract of *C. trichotomum* were measured as the *S. anguivi* and *D. sinuata* did not exhibit free radical scavenging activity when screened with DPPH. Hence, it can be concluded that their traditional uses are not likely to be due to intrinsic free radical scavenging activities although DPPH radical scavenging activity of *S. anguivi* has been reported to be Elekofehinti et al. (2013). The results of the free radical scavenging activities of the selected plants are summarized in table below (Table 4).

**Table 4:** IC_50_ of test samples against DPPH· radical

Plants name	Solvent	IC_50_ (µg/ml)
*C. trichotomum*	Methanol	33 ± 2.4
*P. cablin*	Methanol	23 ± 0.15
*B. platyphylla*	Methanol	12 ± 0.5
Ascorbic acid	Methanol	9 ± 0.5
*C. trichotomum*	Ethanol	67 ± 3.0

Values are mean ± SD three replicates

**Fig. 4:**
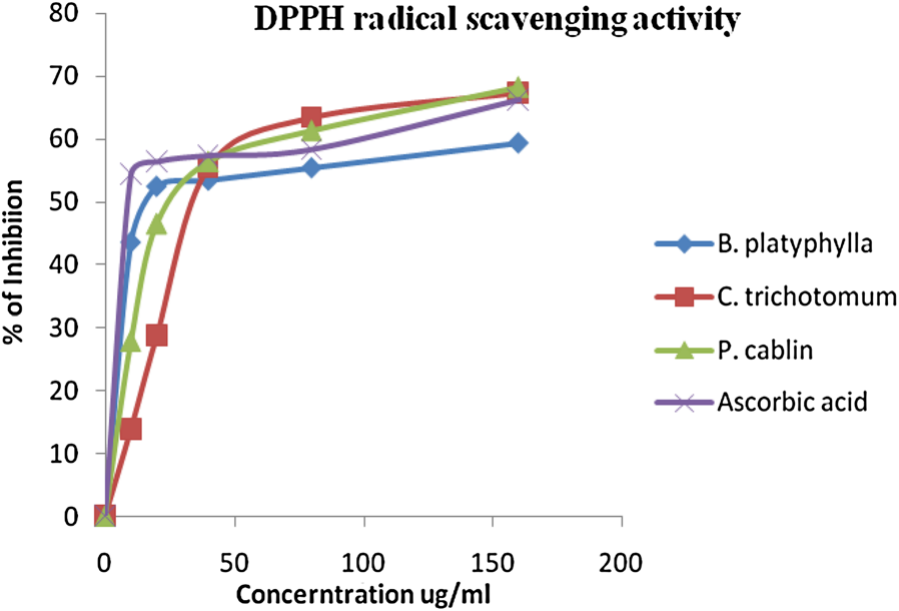
Antioxidant activity of methanol extract of three plants, ascorbic acid on DPPH· radical, results mean ± SD (n = 3)

**Fig. 5:**
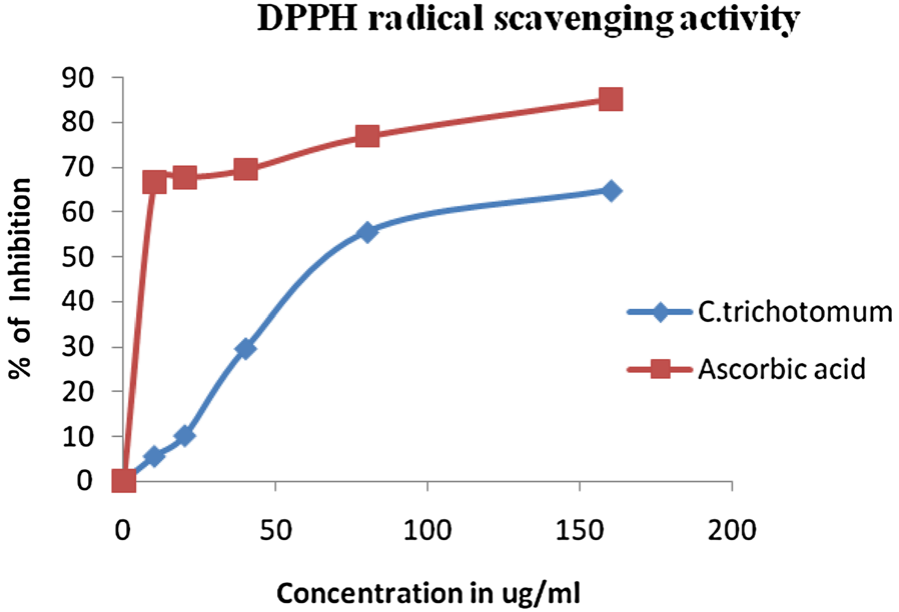
Antioxidant activity of ethanol extract of *C. trichotomum* and ascorbic acid on DPPH· radical, results mean ± SD (n = 3)

The IC_50_ was calculated from the graph obtained by plotting the % scavenging against concentrations used (Figs. 4, 5). The present study shows that almost all the methanol extracts showed potential free radical scavenging capacity. IC_50_ of the extract indicates the corresponding concentration in which the radical scavenging capacity is 50 %. The IC_50_ of the *C. trichotomum*, *P. cablin* and *B. platyphylla* are 33, 23 and 12 µg/ml respectively while IC_50_ value of ascorbic acid (Standard antioxidant) is 9 µg/ml.

Among the methanol extracts tested, *B. platyphylla* displayed a lower IC_50_ value. In comparing the antioxidant activity of methanol and ethanol extract of *C. trichotomum*, methanol extract showed a lower IC_50_ value. This output indicates that the phytochemical is responsible for antibacterial and antioxidant properties of *C. trichotomum* are not same.

#### FRAP assay

**Fig. 6:**
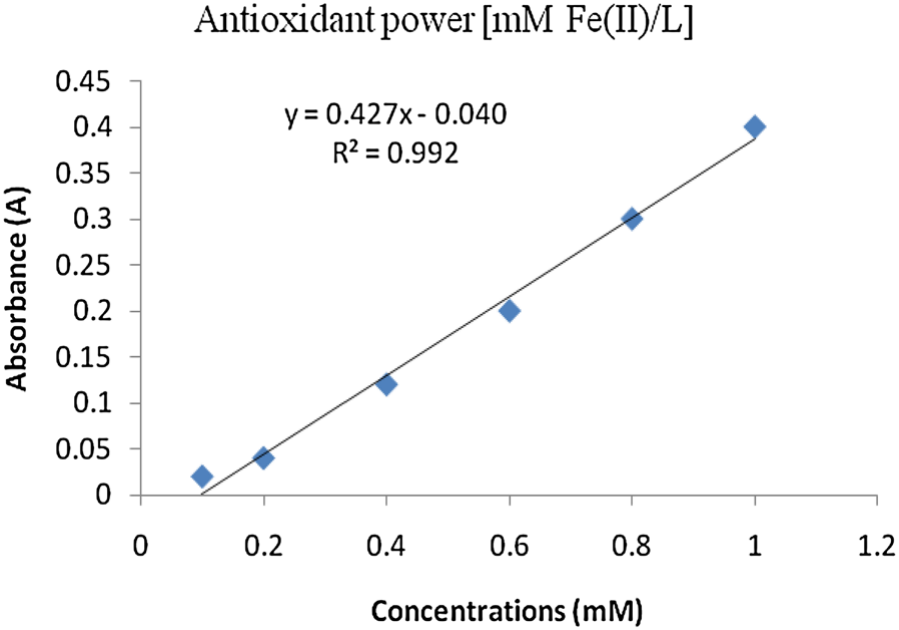
Graph of aborbance versus concentration of ferrous sulphate

The FRAP assay measures the total antioxidant activity on the basis of its ability to reduce a ferric salt Fe(III)(TPTZ)_2_Cl_3_ to Fe(II) ions (Benzie and Strain 1996). The FRAP assay was carried out under acidic conditions (pH 3.6) in order to maintain the iron solubility. With reference to the calibration curve obtained at 593 nm for ferrous sulphate solution (R^2^ = 0.992) (Fig. 6), the FRAP values of methanol extracts of *D. sinuata*, *S. anguivi*, *P. cablin*, *B. platyphylla*, *C. trichotomum* and ethanol extract of *C. trichotomum* were found 140.0, 187.0, 2440.0, 2795.0, 1045.0 and 1811.0 mM Fe(II)/g dry mass (DM) respectively (Table 5).

**Table 5:** Total antioxidant power (FRAP) of test plants extract

Plants name	Solvent	FRAP value mM Fe(II)/gm DM
*D. sinuate*	Methanol	140 ± 2.8
*S. anguivi*	Methanol	187 ± 23
*P. cablin*	Methanol	2440 ± 5.5
*B. platyphylla*	Methanol	2795 ± 108
*C. trichotomum*	Methanol	1045 ± 164
*C. trichotomum*	(Ethnanol)	1811 ± 27

Values are mean ± SD three replicates

*DM* dry mass

Considering the phytochemical screening, DPPH radical scavenging activity and FRAP assay as indices of antioxidant activity of the extract, these findings revealed *B. platyphylla*, *P. cablin*, and *C. trichotomum* as the potential source of natural antioxidants.

Literature review reveals that the phenolic compounds containing free hydrogen are largely responsible for antioxidant activity. Thus the phenolic compounds detected in each of the extract during phytochemical screening can be referred to be responsible for the antioxidant activity. Further, the antioxidant activity also depends upon the quantity of the phytochemicals which is responsible for the hydrogen transfer so as this may be the reason behind different IC_50_ values of different extracts show the presence of polyphenols and flavonoids in the phytochemical screening.

## Conclusions

As the *Yakkha* communities of Chanuwa are having rich knowledge of ethnopharmacology, they have been using it since ages. Several local medicinal plant species in their traditional healthcare delivery system are in practice but they are not used properly. The knowledge that has been transmitted to the generations orally is being extinct due to the introduction of modern medical facilities in the district. Therefore, there is a probability of declination of such traditional medicine practices in the days to come. Hence, such knowledge demands for proper documentation, which can be helpful for screening new active compounds in future. Here it is concluded that there are higher antioxidant properties for the plant *B. platyphylla* and *P. cablin*, and higher antimicrobial activity is for *C. trichotomum* among the plants tested. The most active extracts can be subjected to isolation of the active components and carry out further pharmacological evaluation. This report complements the previously reported curative values and it also highlights an urgency for the further investigations of these pharmaceutically relevant plants.

## Methods

### Collection and processing of plants

Ethnopharmacological data were collected from Chanuwa VDC ward no. 8 of Dhankuta district from July to September 2013. While collecting information on ethno medico botanical aspects, from old aged *Yakkha* people, local professional health healers (Dhamis), standard approaches and methodologies have been followed. The collected specimens were critically studied and identified with the help of available literature (Hara and Williams 1979; Hara et al. 1978). The specimens were further tallied with the voucher specimens deposited as National Herbarium and Plant Laboratories, Godavari (KATH). Extraction was carried out by soaking 150 g of dried powdered samples in about 600 ml of methanol (Analar grade) for 3 days. The extracts were filtered first through cotton wool, then through Whatman filter paper no. 42 (125 mm). The collected extract was dried using a rotary evaporator.

### Chemicals

The chemicals used were methanol (Merck, Germany), DPPH, TPTZ and Ascorbic acid (Sigma Aldrich, USA). All other chemicals used were of the commercially available highest grade.

### Phytochemical screening

The method employed for the phytochemical screening was based on the procedure given by Prof. Culie (1982). It is the process by which the presence of main groups of natural constituents in different extracts is analyzed by using different specific reagents.

### Antibacterial screening

Inhibition of bacterial growth was tested by using the paper disc diffusion method with slight modification as given below (Bauer et al. 1966).

### Microorganism

The micro-organisms used in this study were identified strains obtained from Central Department of Microbiology, TU, Nepal. Among bacteria taken in this study, two were gram positive and four were gram negative as given below.

### Gram positive bacteria

*Staphylococcusaureus* and *Bacillus subtilis.*

### Gram negative bacteria

*Escherichia coli*, *Proteus vulgaris*, *Salmonella typhi* and *Klebsiella pneumoniae.*

### Antimicrobial assay

The antimicrobial activity of the plant extracts were carried by disc diffusion method (Bauer et al. 1966). A suspension of tested micro organisms was spread on Muller-Hilton Agar (MHA) medium. The sterile filter paper discs (6 mm in diameter) were individually impregnated with different concentration of plant extract prepared in dimethyl sulphoxide (DMSO) and then placed into the agar plates which had previously been inoculated with the tested micro organisms. Concentration of the extracts employed was 10 mg/ml. The plates were subsequently incubated overnight at 37 °C. After incubation the growth inhibition rings were quantified by measuring the diameter of the zone of inhibition in mm. For control dimethyl sulphoxide (DMSO) discs were used. All tests were performed in triplicate.

### Antioxidant activity

#### DPPH radical scavenging activity (RSA) assay

The free radical scavenging activity of samples and standard ascorbic acid solution in methanol and ethanol were determined based on their ability to react with stable 1,1-diphenyl-2-picrylhyrazyl (DPPH) free radical (Brand-williams et al. 1995; Blois 1958). The plant samples at various concentrations (15–250 µg/ml) were added to a 100 µM solution of DPPH in methanol. After incubation at 37 °C for 30 min, the absorbance of each solution was determined at 517 nm. The measurement was performed in triplicates. The antioxidant activity of the samples was expressed as IC_50_ (inhibitory concentration), which was defined as the concentration (in µg/ml) of sample required to inhibit the formation of DPPH radicals by 50 %. Ascorbic acid was used as positive control. Free radical scavenging activity was calculated by using following equation:

$$\% \;{\text{of}}\;{\text{free}}\;{\text{radical}}\;{\text{scavenging}}\;{\text{activity}} = \frac{{\left( {{\mathbf{A}}_{0} - {\mathbf{A}}_{{\mathbf{T}}} } \right) \times {\mathbf{100}}}}{{{\mathbf{A}}_{0} }}$$

where A_0_ = absorbance of DPPH solution and A_T_ = absorbance of test or reference sample. The % scavenging was then plotted against concentrations used and from the graph IC_50_ was calculated.

#### FRAP assay

The antioxidant activity by FRAP assay was conducted according to the procedure given by Benzie and Strain (1996). The FRAP reagent was prepared by mixing acetate buffer of pH 3.6 (300 mM), TPTZ (tripyridyltriazine) solution of 10 mM and ferric chloride solution of 20 mM in the ratio of 10:1:1. Antioxidant activity was calculated with the standard calibration of ferrous sulfate. The leaf extracts (5 mg/ml) was prepared by adding methanol and was used as sample. Finally, absorbance was taken at 593 nm keeping the temperature 37 °C.

#### Statistics

All the analysis was carried out in triplicate and the results are expressed as mean ± SD.
